# Identifying most important predictors for suicidal thoughts and behaviours among healthcare workers active during the Spain COVID-19 pandemic: a machine-learning approach

**DOI:** 10.1017/S2045796025000198

**Published:** 2025-05-08

**Authors:** Itxaso Alayo, Oriol Pujol, Jordi Alonso, Montse Ferrer, Franco Amigo, Ana Portillo-Van Diest, Enric Aragonès, Andrés Aragon Peña, Ángel Asúnsolo Del Barco, Mireia Campos, Meritxell Espuga, Ana González-Pinto, Josep Maria Haro, Nieves López-Fresneña, Alma D. Martínez de Salázar, Juan D. Molina, Rafael M. Ortí-Lucas, Mara Parellada, José Maria Pelayo-Terán, Maria João Forjaz, Aurora Pérez-Zapata, José Ignacio Pijoan, Nieves Plana, Elena Polentinos-Castro, Maria Teresa Puig, Cristina Rius, Ferran Sanz, Cònsol Serra, Iratxe Urreta-Barallobre, Ronny Bruffaerts, Eduard Vieta, Víctor Pérez-Solá, Philippe Mortier, Gemma Vilagut

**Affiliations:** 1 Hospital del Mar Research Institute, Barcelona, Spain; 2 Biosistemak Institute for Health Systems Research, Bilbao, Bizkaia, Spain; 3 Red de Investigación en Cronicidad, Atención Primaria y Promoción de la Salud RICAPPS-(RICORS), Instituto de Salud Carlos III (ISCIII), Madrid, Spain; 4 Department of Medicine and Life Sciences (MELIS), Universitat Pompeu Fabra, Barcelona, Spain; 5 Departament de Matemàtiques i Informàtica, Universitat de Barcelona, Barcelona, Spain; 6 CIBER de Epidemiología y Salud Pública (CIBERESP), Instituto de Salud Carlos III, Madrid, Spain; 7 Institut d’Investigació en Atenció Primària IDIAP Jordi Gol, Barcelona, Spain; 8 Atenció Primària Camp de Tarragona, Institut Català de la Salut, Tarragona, Spain; 9 Epidemiology Unit, Regional Ministry of Health, Community of Madrid, Madrid, Spain; 10 Fundación Investigación e Innovación Biosanitaria de AP, Comunidad de Madrid, Madrid, Spain; 11 Department of Surgery, Medical and Social Sciences, Faculty of Medicine and Health Sciences, University of Alcala, Alcalá de Henares, Spain; 12 Ramón y Cajal Institute of Sanitary Research (IRYCIS), Madrid, Spain; 13 Department of Epidemiology and Biostatistics, Graduate School of Public Health and Health Policy, The City University of New York, New York, NY, USA; 14 Service of Prevention of Labor Risks, Medical Emergencies System, Generalitat de Catalunya, Barcelona, Spain; 15 Occupational Health Service, Hospital Universitari Vall d’Hebron, Barcelona, Spain; 16 BIOARABA, Hospital Universitario Araba-Santiago, UPV/EHU, Vitoria-Gasteiz, Spain; 17 CIBER Salud Mental (CIBERSAM), Madrid, Spain; 18 Parc Sanitari Sant Joan de Déu, Institut de Recerca Sant Joan de Deu (IRSJD), Sant Boi de Llobregat, Barcelona, Spain; 19 Hospital General Universitario Gregorio Marañón, Madrid, Spain; 20 UGC Salud Mental, Hospital Universitario Torrecárdenas, Almería, Spain; 21 Villaverde Mental Health Center, Clinical Management Area of Psychiatry and Mental Health, Psychiatric Service, Hospital Universitario 12 de Octubre, Madrid, Spain; 22 Research Institute Hospital 12 de Octubre (i+12), Madrid, Spain; 23 Facultad de Medicina, Universidad Francisco de Vitoria, Madrid, Spain; 24 Servicio de Medicina Preventiva y Calidad Asistencial, Hospital Clínic Universitari de Valencia, Valencia, Spain; 25 Servicio de Psiquiatría y Salud Mental, Hospital el Bierzo, Gerencia de Asistencia Sanitaria del Bierzo (GASBI). Gerencia Regional de Salud de Castilla y Leon (SACYL), Ponferrada, León, Spain; 26 Area de Medicina Preventiva y Salud Pública, Departamento de Ciencias Biomédicas, Universidad de León, León, Spain; 27 National Center of Epidemiology, Instituto de Salud Carlos III (ISCIII), Madrid, Spain; 28 Hospital Universitario Príncipe de Asturias, Servicio de Prevención de Riesgos Laborales, Spain; 29 Clinical Epidemiology Unit-Hospital Universitario Cruces/ OSI EEC, Biobizkaia Health Research Institute, Barakaldo, Spain; 30 Ramón y Cajal University Hospital, IRYCIS, Department of Surgery, Medical and Social Sciences, Faculty of Medicine and Health Sciences, University of Alcalá, Alcala de Henares, MAD, Spain; 31 Research Unit, Primary Care Management, Madrid Health Service, Madrid, Spain; 32 Department of Medical Specialities and Public Health, King Juan Carlos University, Madrid, Spain; 33 Universitat Autònoma de Barcelona (UAB), Barcelona, Spain; 34 Department of Epidemiology and Public Health, Hospital de la Santa Creu i Sant Pau, Barcelona, Spain; 35 Biomedical Research Institute Sant Pau (IIB Sant Pau), Barcelona, Spain; 36 CIBER Enfermedades Cardiovasculares (CIBERCV), Madrid, Spain; 37 Agència de Salut Pública de Barcelona, Barcelona, Spain; 38 Research Progamme on Biomedical Informatics (GRIB), Hospital del Mar Research Institute, Barcelona, Spain; 39 Instituto Nacional de Bioinformatica – ELIXIR-ES, Barcelona, Spain; 40 CiSAL-Centro de Investigación en Salud Laboral, Hospital del Mar Research Institute/University Pompeu Fabra, Barcelona, Spain; 41 Occupational Health Service, Hospital del Mar, Barcelona, Spain; 42 Osakidetza Basque Health Service, Donostialdea Integrated Health Organisation, Donostia University Hospital, Clinical Epidemiology Unit, San Sebastián, Spain; 43 Biodonostia Health Research Institute, Clinical Epidemiology, San Sebastián, Spain; 44 Center for Public Health Psychiatry, Universitair Psychiatrisch Centrum, KU Leuven, Leuven, Belgium; 45 Institute of Neuroscience, Hospital Clinic, University of Barcelona, IDIBAPS, Barcelona, Spain; 46 Institute of Neuropsychiatry and Addiction (INAD), Parc de Salut Mar, Barcelona, Spain

**Keywords:** attempted suicide, interpretability, machine learning, mental health, suicidal ideation

## Abstract

**Aims:**

Studies conducted during the COVID-19 pandemic found high occurrence of suicidal thoughts and behaviours (STBs) among healthcare workers (HCWs). The current study aimed to (1) develop a machine learning-based prediction model for future STBs using data from a large prospective cohort of Spanish HCWs and (2) identify the most important variables in terms of contribution to the model’s predictive accuracy.

**Methods:**

This is a prospective, multicentre cohort study of Spanish HCWs active during the COVID-19 pandemic. A total of 8,996 HCWs participated in the web-based baseline survey (May–July 2020) and 4,809 in the 4-month follow-up survey. A total of 219 predictor variables were derived from the baseline survey. The outcome variable was any STB at the 4-month follow-up. Variable selection was done using an L1 regularized linear Support Vector Classifier (SVC). A random forest model with 5-fold cross-validation was developed, in which the Synthetic Minority Oversampling Technique (SMOTE) and undersampling of the majority class balancing techniques were tested. The model was evaluated by the area under the Receiver Operating Characteristic (AUROC) curve and the area under the precision–recall curve. Shapley’s additive explanatory values (SHAP values) were used to evaluate the overall contribution of each variable to the prediction of future STBs. Results were obtained separately by gender.

**Results:**

The prevalence of STBs in HCWs at the 4-month follow-up was 7.9% (women = 7.8%, men = 8.2%). Thirty-four variables were selected by the L1 regularized linear SVC. The best results were obtained without data balancing techniques: AUROC = 0.87 (0.86 for women and 0.87 for men) and area under the precision–recall curve = 0.50 (0.55 for women and 0.45 for men). Based on SHAP values, the most important baseline predictors for any STB at the 4-month follow-up were the presence of passive suicidal ideation, the number of days in the past 30 days with passive or active suicidal ideation, the number of days in the past 30 days with binge eating episodes, the number of panic attacks (women only) and the frequency of intrusive thoughts (men only).

**Conclusions:**

Machine learning-based prediction models for STBs in HCWs during the COVID-19 pandemic trained on web-based survey data present high discrimination and classification capacity. Future clinical implementations of this model could enable the early detection of HCWs at the highest risk for developing adverse mental health outcomes.

**Study registration:**

NCT04556565

## Introduction

Suicide is a major public health issue and one of the leading causes of preventable death worldwide (World Health Organization, 2024). Pre-pandemic studies showed consistently that both physicians (Dutheil *et al.*, 2019) and nurses (Davis *et al.*, 2021) are at high risk for suicide compared to other employed people (Milner *et al.*, 2013), in part related to high access to lethal means and low willingness to seek help (Harvey *et al.*, 2021). An important risk factor for suicide is suicidal thought and behaviour (STB) (Ribeiro *et al.*, 2016). Studies carried out during the pandemic found high levels of STBs among healthcare workers (HCWs) compared to the pre-pandemic period (Greenberg *et al.*, 2020; Mediavilla *et al.*, 2023; Mortier *et al.*, 2021b; Murata *et al.*, 2021; Sahimi *et al.*, 2021; Xiaoming *et al.*, 2020; Xu *et al.*, 2021; Zhou *et al.*, 2020).

Risk factors identified in these studies span various risk domains and include pre-pandemic lifetime factors, current mental disorders and emotional problems (e.g., burn-out, traumatic stress, anxiety and depression), loneliness and social isolation, financial stress, and pandemic-specific factors, such as having been in quarantine, moral injury, interpersonal and health-related stress (Eyles *et al.*, 2021; García-Iglesias *et al.*, 2022; Mortier *et al.*, 2022, 2021a).

Identifying individuals at the highest risk for future STBs is a significant challenge in the field of mental health research, especially given the relatively low occurrence of STBs. Over the past five decades, traditional statistical approaches have been predominant in predicting STB risk (Nordin *et al.*, 2023), which often, due to their limited capacity as to including a wide range of predictor variables, require the researcher to define a priori a limited set of predictors to be included in the models. This approach has been criticized because variable selection is often based on predefined theoretical frameworks that only consider some of the potentially relevant predictors for STBs (Franklin *et al.*, 2017), resulting in relatively simple models with limited predictive accuracy (Boudreaux *et al.*, 2021).

Advanced analytical approaches, such as machine learning (ML) models, have demonstrated higher predictive accuracy of STBs than traditional statistical approaches (e.g. linear regression, generalized linear models or analysis of variance) including a limited number of variables based on predefined theoretical frameworks (Schafer *et al.*, 2021). ML models handle complex interactions and high-dimensional data effectively by capturing non-linear relationships and efficiently processing and analysing large volumes of data with multiple variables, overcoming the limitations of traditional approaches (Bennett *et al.*, 2022). They also allow a better understanding of the complex patterns and non-evident relationships among a very large set of STB-related variables including not only commonly considered factors such as mental health and family history, but also contextual aspects such as lifestyles, access to healthcare, adverse childhood experiences and social and economic environments, among others (Favril *et al.*, 2022). Thus, these advanced approaches can provide a more comprehensive and accurate understanding of the factors contributing to the risk of STBs. The key is to integrate traditional approaches with the empirical power of data-driven techniques (Schafer *et al.*, 2021). An increased focus on the prediction of adverse mental health, including identification of predictors that mostly contribute to increased prediction accuracy, may lead to new hypotheses about causal associations, and ultimately, a better understanding and prevention of these outcomes, including STBs (Yarkoni and Westfall, 2017).

Despite ML being increasingly used for STB risk prediction, to the best of our knowledge, there is no previous study using ML to develop a prediction model for STBs among HCWs. Such models could help with early identification and intervention for at-risk HCWs and also provide valuable insights into the complex interplay of factors contributing to suicidal ideation in this population. In addition, although there are clear gender differences in both absolute STB risk and the distribution of risk factors (Gradus *et al.*, 2021; Jiang *et al.*, 2021; Miranda‐Mendizabal *et al.*, 2019a, 2019b), ML-based studies often do not take these differences into consideration, leading to a lack of gender-specific STB prediction models.

The aim of the current study is to develop an ML-based prediction model for future STBs using data from the MINDCOVID project, a large prospective cohort study of Spanish HCWs (Alonso *et al.*, 2021; MINDCOVID, 2020). The HCW cohort was recruited just after the height of the first wave of the Spanish COVID-19 pandemic and was followed up 4 months later, including a reassessment of STBs. Importantly, predictor variables to develop the prediction model were created using all information included in the baseline survey. Although the information collected in the baseline survey was not exhaustive with regard to including all factors potentially related to HCW’s STB in the literature, it spanned various relevant risk factor domains for adverse mental health and STB, including depression, anxiety and post-traumatic stress disorder (PTSD). Variable selection techniques were employed to avoid manual selection of predictors. In addition, we aim to identify predictors that are the most important contributors to the model’s predictive accuracy, separately for men and women.

## Methods

### Recruitment

Data for this study come from the MINDCOVID project (Alonso *et al.*, 2021; MINDCOVID, 2020), a multicentre, prospective, observational cohort study of Spanish HCWs, representing a convenience sample of 18 healthcare institutions (hospitals, primary care and public healthcare centres) from six Autonomous Communities in Spain and included all types of HCWs (medical doctors, nurses, auxiliary nurses, other professions involved in patient care and professions not directly involved in patient care). The cohort was assessed at two time points using web-based self-report surveys. The first assessment (T1) was conducted from 5 May through 7 September 2020, i.e., just after the height of the first wave of the Spain COVID-19 pandemic. The follow-up assessment (T2) was conducted 4 months (mean = 120.1 days [SD = 22.2]) after the T1 assessment.

Recruitment for the T1 survey was done by healthcare representatives who contacted all employed HCWs in each participating healthcare centre using administrative email distribution lists (i.e., census sampling). A total of 8,996 HCWs participated at T1, representing a mean weighted response rate across healthcare centres (weighted by achieved sample size) of 11.7% (unweighted mean response rate of 12.8%). A total of 4,809 T1 participants also participated at T2 (53.5%). For both surveys, two reminder emails were sent within 2–4 weeks after the initial invitation. For the current study, we included data from the 4,809 HCWs described previously (Alonso *et al.*, 2022; Mortier *et al.*, 2022) that participated in both T1 and T2 assessments.

Informed consent was obtained from all participants. The study complies with the principles established by national and international regulations, including the Declaration of Helsinki and the Code of Ethics. The study was approved by the Research Integrity and Good Scientific Practices Committee of IMIM‐Parc de Salut Mar, Barcelona, Spain (2020/9203/I), and by all participating centres’ institutional review boards.

### Measures

#### Primary outcome

The study’s primary outcome was any 30-day STBs at the 4-month follow-up (T2), assessed using a modified version of four selected items from the Columbia Suicide Severity Rating Scale (Posner *et al.*, 2011), each with dichotomous response options (yes or no). The items assess passive suicidal ideation (SI) (‘wish you were dead or would go to sleep and never wake up’), active SI (‘have thoughts of killing yourself’), suicide plans (‘think about how you might kill yourself [e.g. taking pills, shooting yourself] or work out a plan of how to kill yourself’) and suicide attempts in the past 30 days (‘make a suicide attempt [i.e. purposefully hurt yourself with at least some intent to die]’). Following previous studies, the primary outcome labelled as ‘any STB’ was created as a dichotomous variable indicating the presence of any of the four STB outcomes (Mortier *et al.*, 2021a; Nock *et al.*, 2014).

#### Baseline predictor variables

The baseline survey (T1) contains 207 items that were used to create the 219 predictor variables for STBs (items with non-ordinal categorical answers were converted into as many dummy variables as the number of categories minus one). The items were organized into eight different sections based on their contents (see Supplementary Table 1 for the list of items). Due to space constraints, we provide here a short description of predictor variables and corresponding T1 survey sections: (1) eight sociodemographic variables (e.g., age, gender and marital status); (2) 14 variables related to COVID-19 exposure, infection status and perceived risk for COVID-19 infection (e.g., having received a positive COVID-19 test and having been hospitalized for COVID-19); (3) 55 items related to mental disorders, including a checklist for pre-pandemic lifetime mental disorders and screening scale items spanning five common current mental disorders, i.e., Major Depressive Disorder (PHQ-8; Kroenke *et al.*, 2009), Generalized Anxiety Disorder 7-item (Spitzer *et al.*, 2006), 30-day panic attacks (item adapted from the Composite International Diagnostic Interview (CIDI) screening scale; Kessler *et al.*, 2013), 30-day traumatic stress symptoms (four-item abbreviated form of the PTSD Checklist, PCL-5; Zuromski *et al.*, 2019) and substance use disorder (four-item version of the CAGE Adapted to Include Drugs (CAGE-AID); Hinkin *et al.*, 2001). In addition, any 30-day STB was assessed (Posner *et al.*, 2011), as well as burnout (six-item personal burnout subscale of the Copenhagen Burnout Inventory; Kristensen *et al.*, 2005), 30-day psychotic symptoms (items taken from the prodromal questionnaire; Loewy *et al.*, 2011) and 30-day obsessive compulsive disorder symptoms (the three-item obsessing subscale of the obsessive compulsive inventory revised; Foa *et al.*, 2002); (4) 30 items assessing treatment use, including healthcare service and psychotropic medication use for emotional or substance use problems, as well as barriers for treatment use; (5) 27 items assessing relevant work-related variables (e.g., type of HCWs, type of workplace, income, perceived risk for COVID-19 at work, perceived lack of healthcare centre preparedness and moral injury); (6) three items about isolation, quarantine and confinement due to COVID-19; (7) 35 items assessing 12-month serious stressful events, perceived stress (adapted peri life events scale; Dohrenwend *et al.*, 1978), resilience (Connor–Davidson resilience scale; Connor and Davidson, 2003) and healthy habits and (8) 35 items assessing social support (Oslo social support scale; Husain *et al.*, 2016), loneliness (UCLA three-item loneliness scale; Hughes *et al.*, 2004), use of social media, family functioning (Brief Assessment of Family Functioning Scale; Mansfield *et al.*, 2019), parental stress (items taken from the Parental Stress Scale; Berry and Jones, 1995), quality of life (five-level version of EQ-5D; Herdman *et al.*, 2011), somatic comorbidity (self-administered comorbidity questionnaire (Sangha *et al.*, 2003) and role impairment (Sheehan disability scales; Sheehan *et al.*, 1996).

#### Statistical analysis

The percentage of missing values across all variables analysed was moderate, with a mean missing rate of 6.5% and a median value of less than 1% (see Supplementary Table 2 for the percentage of missing values for each variable). Multiple imputation by chained equations with 10 iterations per imputation and 12 imputed datasets was used to impute missing item-level data (Van Buuren, 2018) using R’s mice package (Buuren and Groothuis-Oudshoorn, 2011). The choice of 12 imputations provided a reasonable trade-off between statistical accuracy and computational efficiency, following recommendations that 5–20 imputations are generally sufficient under moderate missingness (Van Buuren, 2018; White *et al.*, 2011) and that the number of imputations should be at least equal to 100 times the fraction of missing information, which in our study was below 0.1 (White *et al.*, 2011) for key performance measures such as AUC.

The regularization path of linear Support Vector Classifier (SVC) with L1 penalty (Dai and Zhao, 2020) was implemented to select the most critical predictor variables out of the 219 candidates by forcing some coefficients to be exactly zero, aiming to improve the accuracy and efficiency of predictive models (Montesinos López & Crossa, 2022). SVC for variable selection was applied to the 12 imputed datasets. Variables that were selected in at least 7 of the 12 imputed datasets were included in the final prediction model. This decision is justified by the work of Zhao and Long (Zhao and Long, 2017) who propose to perform variable selection separately in each imputed dataset and then include variables that are selected with a frequency above a defined threshold. In this study, the threshold chosen was 7 out of 12 imputations to ensure consistency across more than 50% of the imputations. According to Wood *et al.* (2008), this strategy improves the robustness of variable selection and coefficient estimation in regression models.

To address imbalanced data (i.e., 7.9% prevalence of STBs at the 4-month follow-up), which can lead to poor minority class classification (Rezvani and Wang, 2023), two different techniques were compared: (1) Synthetic Minority Oversampling Technique (SMOTE) (Chawla *et al.*, 2002) and (2) majority class undersampling (Devi *et al.*, 2021).

A random forest (RF) classifier was used to develop prediction models for STBs at the 4-month follow-up (Hammelrath *et al.*, 2023; Navarro *et al.*, 2021). Different values of the predefined hyperparameters specifying the number of decision trees to be included in the RF (n_estimators: [20, 25, 50, 75, 100]) and the maximum depth allowed for each decision tree (max_depth: [7, 8, 9, 10, 11]) were tested using a grid combination and 5-fold cross-validation. The grid combination was tested on a single stacked dataset of all 12 imputed datasets for both balancing methods (Seki *et al.*, 2021). The model with the selected hyperparameters was then independently trained and tested for each of the 12 imputed datasets. To decrease the risk of overfitting, 5-fold cross-validation was used in each imputed dataset. We aggregated the results of the predicted probabilities of each of the imputations into a single dataset to obtain performance metrics. The RandomForestClassifier function of the sklearn library in Python version 3.8 was used (Pedregosa *et al.*, 2012).

Model characteristics were assessed on the test dataset through the area under the Receiver Operating Characteristic (AUROC) curve and the area under the precision (positive predictive value [PPV])–recall (sensitivity) curve. The precision–recall curve is particularly useful for imbalanced datasets (Saito and Rehmsmeier, 2015). These curves allowed us to evaluate recall, specificity and precision for different cut-off points. The model was applied to each of the imputed datasets and predictions from each dataset were aggregated to obtain the overall metrics values (Seki *et al.*, 2021). All metrics were also obtained separately for men and women.

To quantify variable importance, the Shapley Additive Explanations (SHAP) (Lundberg and Lee, 2017) method was used. SHAP values represent the contribution that each variable had in the final model prediction. As SHAP values can display variability across imputations, they were obtained separately for each of the 12 imputed datasets and a combined representation of the contributions of each variable was obtained as the mean of these values (Seki *et al.*, 2021). Although the model is the same for both genders, the SHAP values have been obtained separately for the subsamples of men and women. SHAP summary plots are provided. In this plot, variables are ordered according to their influence on the predictions of the model. Each dot represents an individual’s SHAP value, plotted along the horizontal axis. The dots are collared based on the variable’s value, ranging from low (blue) to high (pink). If pink dots appear on the right side and blue dots on the left, it indicates that the risk increases as the value of the variable rises. Analyses were conducted with the SHAP library in Python version 3.8.

## Results

### Sample characteristics

Table 1 shows the descriptive characteristics of the study sample by gender at T1. Ages ranged from 18 to 71 years, with a mean age of 45.8 (SD = 11.0). The country of birth was Spain for 95.3% of the sample. Healthcare professionals were mostly women (81.1%). About one-third (34.3%) were physicians, 29.2% nurses, 8.1% auxiliary nurses, 11.5% other profession involved in patient care and 16.9% other profession not involved in patient care. A total of 19.5% had a positive test or medical diagnosis of COVID-19 (women = 19.3%, men = 20.2%). A total of 381 subjects (7.9%; women = 7.8%, men = 8.2%) reported having had 30-day STBs at T2.

**Table 1.** Sociodemographic and work characteristics of Spanish healthcare workers during the COVID-19 pandemic assessed at T1 (*N* = 4,809)

	Total (*N* = 4,809)		WOMEN (*n* = 3,988)		MEN (*n* = 910)	
	*n*	% (SE) or mean (SD)	*n*	% (SE) or Mean (SD)	*n*	% (SE) or Mean (SD)
Women	3,899	81.1 (0.8)	–	–	–	–
Age		45.8 (11.0)		45.5 (11.0)		47.1 (11.25)
Country of birth						
Spain	4,582	95.3 (0.4)	3,717	95.3 (0.5)	864	95.0 (0.6)
Other	227	4.7 (0.4)	182	4.7 (0.5)	46	5.0 (0.6)
Living with partner	3,465	72.1 (1.2)	2,764	70.9 (1.3)	701	77.0 (1.2)
Marital status						
Single	1,756	36.5 (3.1)	1,452	37.2 (3.3)	304	33.4 (2.9)
Married	2,537	52.7 (2.6)	2,007	51.5 (2.9)	530	58.2 (1.7)
Divorced or legally separated	459	9.6 (0.5)	387	9.9 (0.4)	72	7.9 (1.2)
Widowed	57	1.2 (0.2)	53	1.4 (0.2)	4	0.4 (0.3)
Children in care	1,995	41.5 (0.9)	1,631	41.8 (0.9)	364	40.0 (1.7)
Healthcare profession						
Physician	1,650	34.3 (4.3)	1,217	31.2 (4.7)	433	47.6 (3.4)
Nurse	1,406	29.2 (1.8)	1,261	32.3 (2.2)	145	15.9 (1.0)
Auxiliary nurse	387	8.1 (1.9)	341	8.7 (2.1)	46	5.1 (2.0)
Other profession involved in patient care	555	11.5 (0.9)	441	11.3 (0.9)	114	12.5 (1.4)
Other profession not involved in patient care	812	16.9 (1.8)	640	16.4 (1.7)	172	18.9 (2.7)
Type of workplace						
Hospital	2,818	58.6 (13.2)	2,273	58.3 (13.4)	545	59.9 (12.4)
Primary care	1,581	32.9 (14.3)	1,313	33.7 (14.5)	268	29.5 (13.6)
Others	410	8.5 (2.6)	313	8.0 (2.4)	97	10.7 (3.3)
Had close contact with someone who has died from COVID-19 because of work	2,190	45.5 (3.3)	1,781	45.7 (3.3)	409	44.9 (4.1)
Positive test or medical diagnosis of COVID-19	936	19.5 (2.2)	752	19.3 (2.1)	184	20.2 (2.9)

SE: standard error; SD: standard deviation.

Of the 7.9% of subjects with any STB at the 12-month follow-up, 0.9% reported a suicide attempt, 26.3% a suicide plan without attempt, 10.7% active ideation without plan or attempt and 62.1% passive ideation without active ideation, plan or attempt.

### Variables selection

Out of the initial 219 candidate predictor variables, 34 variables were selected by the linear SVM (Supplementary Figure 1). Although the gender variable was not selected, it was included because gender is a key variable due to significant differences in STB risk factors between men and women (Miranda-Mendizabal *et al.*, 2019; Schrijvers *et al.*, 2012), and to be able to assess relevant risk factors within each gender. This leads to a total of 35 variables being included in the model. The inclusion in the RF model is also justified by the ability of the RF model to account for complex interactions between variables (Auret and Aldrich, 2012).

### Random forest

The selected hyperparameters specified 50 decision trees to be included in the RF (n_estimators = 50) and a maximum depth of 9 allowed for each decision tree (max_depth = 9).

**Figure 1.**
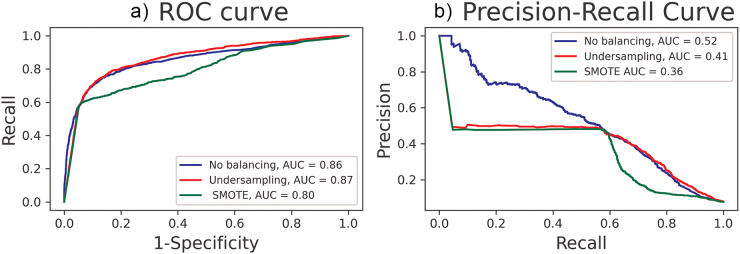
(a) The receiver operating characteristics curve and the area under the receiver operating characteristic curve (AUROC) for suicidal thoughts and behavior prediction. The results of the prediction using different balancing test are shown (left). (b) The precision-recall curve and the area underthe precision-recall curve of the models. The results of the prediction using different balancing test are shown (right).

Abbreviations: ROC: receiver operating characteristics; AUC: area under the curve; No balancing: no balancing technique was used; Undersampling: undersampling of the majority class technique was used; SMOTE: Synthetic Minority Oversampling Technique was used.

Figure 1 presents AUROC and precision–recall curves resulting from the RF fitted on the test sample using the 35 baseline selected variables. The AUROC with and without balancing techniques is higher than 0.80. In the total sample, the best result was obtained with the model without data balancing (AUROC = 0.87).

Regarding the area under the precision–recall curve, large differences are observed between balancing methods, being the model without data balancing the one with the best result (area under the precision–recall curve = 0.52).

**Figure 2.**
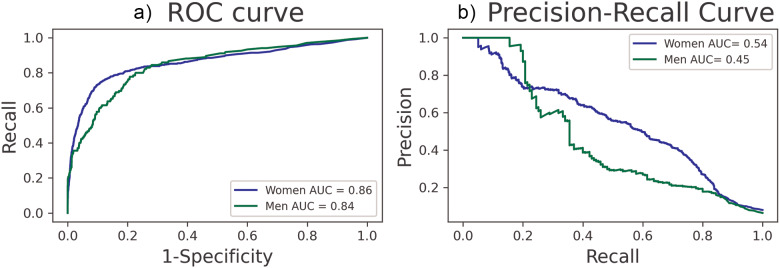
(a) The receiver operating characteristics curve and the area under the receiver operating characteristic (AUROC) curve of the models for men and women. (b) The precision-recall curve and the area under the precision-recall curve of the models for men and women.

Abbreviations: ROC: receiver operating characteristics; AUC: area under the curve.

Figure 2 shows that when the goodness of fit of the model without data balancing is assessed separately by gender, the good metric properties are maintained. The AUROC curve is 0.84 and 0.86 for men and women, respectively. In the case of the area under the precision–recall curve, the values obtained are 0.45 for men and 0.54 for women.

### SHAP values

**Figure 3.**
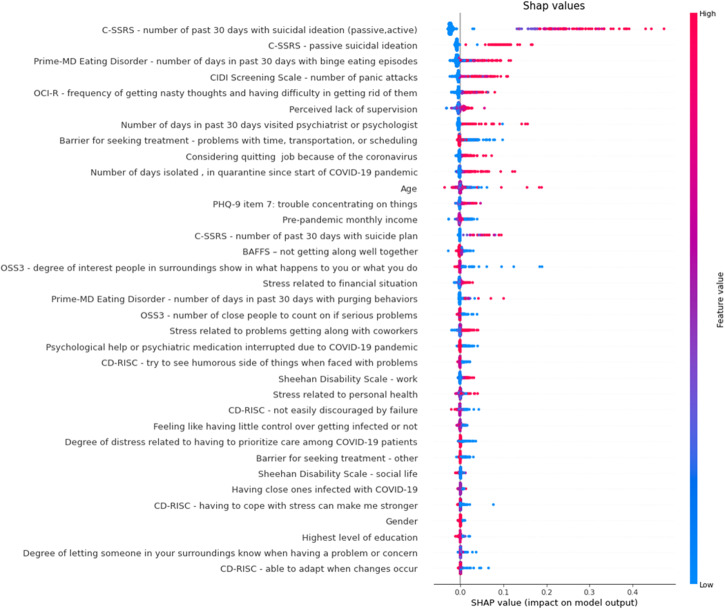
Shapley additive explanation (SHAP) summary graph. Each point on the graph is a SHAP value for one variable. The color represents the value of the variable from low (blue) to high (pink).

*Notes:* The color of each point on the graph represents the value of the corresponding variable: pink indicates high values and blue indicates low values. The horizontal axis (x-axis) represents the SHAP value. Abbreviations: C-SSRS: Suicidal thoughts and behaviors screen; PHQ-9: Patient Health Questionnaire 9 item; CIDI: Composite International Diagnostic Interview; Prime-MD: Primary Care Evaluation of Mental Disorders; BAFFS: Brief Assessment of Family Functioning Scale; CD-RISC: Connor-Davidson Resilience Scale; OSS3: Oslo Social Support Scale; OCI-R: Obsessive Compulsive Inventory revised.

**Figure 4.**
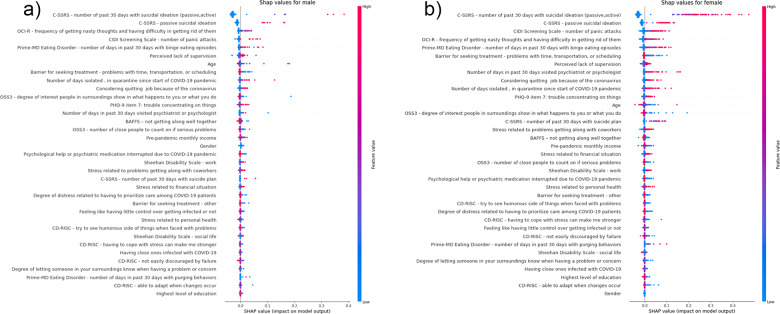
Shapley additive explanation (SHAP) summary graph. for men (a) and women (b).

*Notes:* The color of each point on the graph represents the value of the corresponding variable: pink indicates high values and blue indicates low values. The horizontal axis (x-axis) represents the SHAP value. Abbreviations: C-SSRS: Suicidal thoughts and behaviors screen; PHQ-9: Patient Health Questionnaire 9 item; CIDI: Composite International Diagnostic Interview; Prime-MD: Primary Care Evaluation of Mental Disorders; BAFFS: Brief Assessment of Family Functioning Scale; CD-RISC: Connor-Davidson Resilience Scale; OSS3: Oslo Social Support Scale; OCI-R: Obsessive Compulsive Inventory revised.

In the summary plot of the SHAP values (Figures 3 and 4), the variables are sorted according to their importance in the prediction model. The colour of each point on the graph represents the value of the corresponding variable: pink indicates high values and blue indicates low values. The horizontal axis (*x*-axis) represents the SHAP value: having values above 0 indicates that these experiences are potentially important predictor variables in predicting future suicidal ideation. Figure 3 shows the most important variables in the prediction of STBs. The ranking is headed by the number of days in the past 30 days with suicidal ideation (passive or active) followed by passive suicidal ideation, the number of days in the past 30 days with binge eating episodes and having intrusive thoughts (i.e., nasty thoughts and having difficulty in getting rid of them). Another relevant factor identified is concentration problems. Some of the factors associated with COVID-19 infection that have been identified include: having been in isolation or quarantine, fear of personal or loved ones’ infection, work-related factors and experiences during the initial pandemic outbreak, such as perceived lack of supervision at work, not getting along with co-workers, stress related to having to prioritize care among patients and work-related role impairment. Financial stress also appears as a risk factor for STBs.

Among men (Figure 4a) the most important variables for predicting STBs at T2 included the number of days in the past 30 days with suicidal ideation (passive or active), passive suicidal ideation and frequency of intrusive thoughts at the T1 assessment.

Among women (Figure 4b), the most important baseline variables for predicting STBs (at T2) included the number of days in the past 30 days with suicidal ideation (passive or active), passive suicidal ideation and number of panic attacks.

## Discussion

In this study, we developed and validated a predictive model for STBs within a 4-month period using survey data collected from HCWs during the COVID-19 pandemic in Spain. The ML-based model showed robust predictive performance for STBs and identified, out of a total of 219 variables, the 35 key predictive variables associated with STBs. Our model showed very good metric characteristics with an AUROC of 0.86 (0.86 in women and 0.84 in men) and an area under the precision–recall curve of 0.52 (0.54 and 0.45 in women and men, respectively). The results align with previous studies, as shown in the systematic review by Somé *et al.* (2024), which found a mean AUROC of 0.81 in 84 studies, a mean recall of 0.68 in 64 studies and a mean precision of 0.41 in 46 studies. Our results improve precision metric (Nock *et al.*, 2022), which is challenging due to the low prevalence of STBs. ML models developed to predict STBs in previous studies have been criticized for having low precision (often below 1%) and thus producing too many false positives (Nock *et al.*, 2022). Our model achieved a precision of 50% with a recall of 60%. With recalls of 80%, the precision is greater than 20%. The fact that the model’s cut-off point is not predetermined allows for their selection based on the required recall and precision, depending on the objective or application of the predictive models. As the data were unbalanced (92.1% of the subjects in one category), balancing techniques were tested, but these techniques did not improve the results of the models.

STB at T1 was identified as a strong predictor at T2, consistent with previous literature (Ribeiro *et al.*, 2016). While the association between mental disorders and suicidal ideation is well established (Franklin *et al.*, 2017), a key contribution of our study is the identification of specific mental disorder symptoms as independent predictors of STBs among HCWs active during the COVID-19 pandemic, including binge eating, panic attacks, intrusive thoughts and concentration problems. These results align with studies in non-HCW populations linking STBs to eating disorders (Brown *et al.*, 2018; Sohn *et al.*, 2023), panic disorder (Zhang *et al.*, 2022), obsessive-compulsive disorder (Pellegrini *et al.*, 2021) and concentration difficulties (Lo *et al.*, 2023). These findings highlight the critical need for early identification and screening of mental health symptoms in HCWs, as well as the challenge of ensuring access to timely, evidence-based mental health care (Jain *et al.*, 2024). Notably, our study also found that practical barriers to seeking treatment and disruptions in psychiatric or psychological care due to the COVID-19 pandemic were significant predictors of future STBs, a particularly concerning issue given the low treatment utilization among HCWs (Braquehais *et al.*, 2022; Dellazizzo *et al.*, 2021; Mortier *et al.*, 2024; Rogoža *et al.*, 2021).

Consistent with prior research (Du *et al.*, 2023; Kavukcu and Akdeniz, 2021), our study found that COVID-19-related experiences, including isolation or quarantine and fear of personal or familial infection, predicted STBs at follow-up, likely due to their traumatic and stressful nature (Portillo-Van Diest *et al.*, 2023). Additionally, work-related disruptions during the initial pandemic outbreak, such as perceived lack of supervision, interpersonal conflicts with co-workers, stress from prioritizing patient care and role impairment, emerged as significant predictors of later suicidal ideation. These findings underscore the need for systemic workplace reforms, including improved healthcare centre preparedness for viral outbreaks through enhanced equipment, staffing, training and protocols. Moreover, fostering supportive work environments, encouraging the reporting of interpersonal conflicts (Alshammari and Dayrit, 2017) and implementing effective communication and conflict resolution strategies (Jerng *et al.*, 2017) are essential. Future research should focus on delineating causal pathways underlying STBs among HCWs to inform targeted prevention efforts, addressing the critical gap in evidence-based interventions for mental health issues in this population at both individual and organizational levels (Petrie *et al.*, 2019).

Gender was not selected as a relevant predictor when the Support Vector Machine (SVM) model was applied. This result was unexpected as gender has been shown in the mental health and STB literature to be a key variable in identifying significant risk factors. The ability of the RF model to capture complex interactions, identify non-linear dependencies and consider multivariate relationships between variables (Auret and Aldrich, 2012), together with the recognized clinical and theoretical relevance of gender in mental disorders and STB – given that risk factors differ significantly between men and women (Miranda‐Mendizabal *et al.*, 2019; Schrijvers *et al.*, 2012) – justifies the inclusion of the gender variable in the model.

Considering the evident gender differences in both the absolute risk of STBs and the prevalence of associated risk factors (Gradus *et al.*, 2021; Jiang *et al.*, 2021; Miranda‐Mendizabal *et al.*, 2019a, 2019b), the accuracy of the model was assessed separately for men and women, and the model proved to be a good fit for both genders. For both genders, the most important factors were the number of suicidal thoughts (passive or active) and passive suicidal thoughts in the last 30 days. For men, the second most important factor was the frequency of unpleasant thoughts and the difficulty in getting rid of them, while for women it was the number of panic attacks. This is an important finding suggesting that there are gender differences in the relative importance of risk factors for STBs. Previous research has shown significant interactions between gender and certain risk factors for STBs (Miranda‐Mendizabal *et al.*, 2019a). These gender differences in risk factors have been linked to variations in the prevalence of internalizing and externalizing disorders between genders, as well as differences in coping strategies, including the frequency of help-seeking. These differences may be attributed to gender socialization.

### Strengths and limitations

This study has some limitations. First, due to low numbers of suicide plans and suicide attempts at the 4-month follow-up, we operationalized the study outcome as any STB (i.e., having passive or active suicidal ideation with or without plan or attempt), as all four separate outcomes indicate the presence of at least passive suicidal ideation. This is in line with previous work by our group (Mortier *et al.*, 2021a) and others (Benjet *et al.*, 2022; Nock *et al.*, 2022). Second, a convenience sample was used and results need to be validated using external samples. This limitation is partially addressed by obtaining a random and heterogeneous census sample of HCWs from 18 healthcare institutions in six Autonomous Communities. Third, STB is complex and difficult to predict; therefore, psychosocial and environmental factors cannot be easily excluded (Ati *et al.*, 2021). However, the number of variables used to predict was very large, all 219 variables collected in the survey were used. Fourth, although an SVM with an L1 regularization model was used for a large and objective selection of variables, the analysis is based on a predefined survey with a specific number of items. This implies that, although the model allows for a greater inclusion of predictor variables, there is still the limitation of not including all possible variables relevant in the context of STBs. Fifth, we have exclusively used the RF algorithm to predict STBs. Although the predictive capacity of this algorithm was effective in our study, other models may also provide meaningful and complementary results that could improve the accuracy of our results analysis.

## Conclusion

In this study of Spanish HCWs during the COVID-19 pandemic, we have developed a predictive model of the risk of STBs. Our results show that RF ML algorithm has a high prediction performance for STBs (AUROC = 0.86; 0.86 in women and 0.84 in men). Importantly, our study improves the precision compared to previous research. The results generated by the proposed model help to identify and explain risk factors for STBs and contribute to the development of a first comprehensive conceptual framework for understanding STB occurrence in major epidemics and other disasters with high impact on essential workers. The most important predictors contributing to the prediction of suicide ideation in healthcare professionals were ideation frequency in the last 30 days, passive suicidal ideation and the number of days with binge eating episodes in the last 30 days.

## Supporting information

Alayo et al. supplementary material Alayo et al. supplementary material

## Data Availability

The de-identified participant data as well as the study protocol, statistical analysis plan and data dictionaries used for this study are available as from publication and upon reasonable request from the corresponding author (P.M.; pmortier@researchmar.net) as long as the main objective of the data-sharing request is replicating the analysis and findings as reported in this paper.

## Supplementary material

The supplementary material for this article can be found at https://doi.org/10.1017/S2045796025000198.
