# Supplementary material for: Identifying most important predictors for suicidal thoughts and behaviours among healthcare workers active during the Spain COVID-19 pandemic: a machine-learning approach
Source: Epidemiol Psychiatr Sci. 2025 May 8;34:e28. doi: 10.1017/S2045796025000198 (PMC12090031; doi:10.1017/S2045796025000198)
Supplement: Alayo et al. supplementary material [file S2045796025000198sup001.pdf]

Supplementary Table1. Detailed description of the 207 candidate predictor variables for suicidal ideation that were created using the T1 baseline survey.

**Description item**	**Description response options**	**Recall period**
SOCIODEMOGRAPHIC VARIABLES SECTION		
Age	0-110 years	/
Gender	men, women	/
Country of birth	Spain, other	/
Nationality	only Spain, only other than Spain, both	/
Living with partner	yes/no	/
Marital status	single, married, divorced or legally separated, widowed	/
Highest level of education	7 response options ranging from primary education or lower to Master's, postgraduate studies, or doctorate	/
Having children in care	yes/no	/
COVID-19 INFECTION STATUS SECTION		
Positive test or medical diagnosis COVID-19	yes/no	since COVID-19 pandemic
Number of days hospitalized for COVID-19	0-200	since COVID-19 pandemic
Having been hospitalized for COVID-19	yes/no	since COVID-19 pandemic
Ever admitted to intensive care unit for COVID-19	yes/no	since COVID-19 pandemic
Belief to have been infected with COVID-19	yes/no	since COVID-19 pandemic
Having close ones infected with COVID-19	yes/no	since COVID-19 pandemic
Severity of infection of close one most severely infected	5 response options: without symptoms, mild symptoms, severe symptoms but not hospitalized, hospitalized, deceased	since COVID-19 pandemic
Number of days isolated, in quarantine since start of COVID-19 pandemic	0-200	since COVID-19 pandemic
Having been isolated or in quarantine because of COVID-19	yes/no	since COVID-19 pandemic
ADAPTED COVID-19 PERCEIVED RISK SCALE		
Feeling like having little control over getting infected or not	5-point Likert type item: never, rarely, sometimes, almost always, always	since COVID-19 pandemic
Thinking that it would be unlikely to survive if having coronavirus.	5-point Likert type item: never, rarely, sometimes, almost always, always	since COVID-19 pandemic
Being afraid of infecting loved ones with coronavirus.	5-point Likert type item: never, rarely, sometimes, almost always, always	since COVID-19 pandemic

**Description item**	**Description response options**	**Recall period**
Family and friends are worried about getting infected through me	5-point Likert type item: never, rarely, sometimes, almost always, always	since COVID-19 pandemic
COVID-19 GOVERNMENT MEASURES		
Feeling there has been too much concern about COVID-19 pandemic	not at all, a little, somewhat, a lot	since COVID-19 pandemic
MENTAL HEALTH SECTION		
CIDI 3.0 LIFETIME DISORDER SCREENING QUESTIONS		
CIDI 3.0 - reporting prepandemic depression	yes/no	before COVID-19 pandemic
CIDI 3.0 - reporting prepandemic bipolar disorder	yes/no	before COVID-19 pandemic
CIDI 3.0 - reporting prepandemic panic attacks,disorder	yes/no	before COVID-19 pandemic
CIDI 3.0 - reporting prepandemic anxiety issues	yes/no	before COVID-19 pandemic
CIDI 3.0 - reporting prepandemic alcohol use problems,disorders	yes/no	before COVID-19 pandemic
CIDI 3.0 - reporting prepandemic drug use problems,disorders	yes/no	before COVID-19 pandemic
CIDI 3.0 - reporting any other serious prepandemic psychological problem	yes/no	before COVID-19 pandemic
CIDI 3.0 - no prepandemic psychological problems	yes/no	before COVID-19 pandemic
PATIENT HEALTH QUESTIONNAIRE (PHQ-8)		
PHQ-8 item 1: little interest or pleasure in doing things	5-point Likert type item with response options: not at all, several days, more than half the days, nearly every day	past 14 days
PHQ-8 item 2: feeling down, depressed, or hopeless	5-point Likert type item with response options: not at all, several days, more than half the days, nearly every day	past 14 days
PHQ-8 item 3: trouble falling or staying asleep, or sleeping too much	5-point Likert type item with response options: not at all, several days, more than half the days, nearly every day	past 14 days
PHQ-8 item 4: feeling tired or having little energy	5-point Likert type item with response options: not at all, several days, more than half the days, nearly every day	past 14 days
PHQ-8 item 5: poor appetite or overeating	5-point Likert type item with response options: not at all, several days, more than half the days, nearly every day	past 14 days
PHQ-8 item 6: feelings of low self-esteem or failure	5-point Likert type item with response options: not at all, several days, more than half the days, nearly every day	past 14 days

Description item	Description response options	Recall period
PHQ-8 item 7: trouble concentrating on things	5-point Likert type item with response options: not at all, several days, more than half the days, nearly every day	past 14 days
PHQ-8 item 8: psychomotor agitation or retardation	5-point Likert type item with response options: not at all, several days, more than half the days, nearly every day	past 14 days
GENERAL ANXIETY DISORDER 7 ITEM SCALE (GAD-7)		
GAD-7 item 1: feeling nervous, anxious, or on edge	5-point Likert type item with response options: not at all, several days, more than half the days, nearly every day	past 14 days
GAD-7 item 2: not being able to stop or control worrying	5-point Likert type item with response options: not at all, several days, more than half the days, nearly every day	past 14 days
GAD-7 item 3: worrying too much about different things	5-point Likert type item with response options: not at all, several days, more than half the days, nearly every day	past 14 days
GAD-7 item 4: trouble relaxing	5-point Likert type item with response options: not at all, several days, more than half the days, nearly every day	past 14 days
GAD-7 item 5: being restless or feeling keyed up or on edge	5-point Likert type item with response options: not at all, several days, more than half the days, nearly every day	past 14 days
GAD-7 item 6: becoming easily annoyed or irritable	5-point Likert type item with response options: not at all, several days, more than half the days, nearly every day	past 14 days
GAD-7 item 7: feeling afraid as if something awful might happen	5-point Likert type item with response options: not at all, several days, more than half the days, nearly every day	past 14 days
PTSD CHECKLIST FOR DSM-5 (PCL-5) – 4 ITEMS		
PCL-5 - intrusion: suddenly feeling or acting as if the stressful experience were actually happening again	5-point Likert type item with response options: not at all, a little bit, moderately, quite a bit, extremely	past 30 days
PCL-5 - avoidance: avoiding external reminders of the stressful experience	5-point Likert type item with response options: not at all, a little bit, moderately, quite a bit, extremely	past 30 days
PCL-5 - negative alterations in cognition and mood: feeling distant or cut off from other people	5-point Likert type item with response options: not at all, a little bit, moderately, quite a bit, extremely	past 30 days
PCL-5 - alterations in arousal and reactivity: irritable behavior, angry outbursts, or acting aggressively	5-point Likert type item with response options: not at all, a little bit, moderately, quite a bit, extremely	past 30 days
PANIC ATTACKS - CIDI SCREENING SCALE		
CIDI Screening Scale - number of panic attacks	0-200	past 30 days
CAGE-AID QUESTIONNAIRE		
CAGE-AID - cutting down: felt like should cut down drinking or drug use	yes/no	past 30 days
CAGE-AID - annoyance by others: being annoyed by people criticizing drinking or drug use	yes/no	past 30 days

Description item	Description response options	Recall period
CAGE-AID - feelings of guilt: feeling guilty about drinking or drug use	yes/no	past 30 days
CAGE-AID - dependency: needed drink, drugs first thing in the morning (eye-opener)	yes/no	past 30 days
SUICIDAL THOUGHTS AND BEHAVIORS SCREEN (CSSRS – SITBI)		
C-SSRS - passive suicidal ideation	yes/no	past 30 days
C-SSRS - active suicidal ideation	yes/no	past 30 days
C-SSRS - number of past 30 days with suicidal ideation (passive, active)	0-30	past 30 days
C-SSRS - suicide plan	yes/no	past 30 days
C-SSRS - number of past 30 days with suicide plan	0-30	past 30 days
C-SSRS - suicide attempt	yes/no	past 30 days
PERSONAL BURNOUT SUBSCALE OF THE COPENHAGEN BURNOUT INVENTORY (CBI)		
CBI burnout subscale - frequency feeling tired	5-point Likert-type item: never/almost never, seldom, sometimes, often, always	past 30 days
CBI burnout subscale - frequency feeling physically exhausted	5-point Likert-type item: never/almost never, seldom, sometimes, often, always	past 30 days
CBI burnout subscale - frequency feeling emotionally exhausted	5-point Likert-type item: never/almost never, seldom, sometimes, often, always	past 30 days
CBI burnout subscale - frequency thinking: i can't take it anymore	5-point Likert-type item: never/almost never, seldom, sometimes, often, always	past 30 days
CBI burnout subscale - frequency feeling worn out	5-point Likert-type item: never/almost never, seldom, sometimes, often, always	past 30 days
CBI burnout subscale - frequency feeling feel weak and susceptible to illness	5-point Likert-type item: never/almost never, seldom, sometimes, often, always	past 30 days
SELECTED ITEMS FROM THE PRODROMAL QUESTIONNAIRE		
Prodromal Questionnaire - previously familiar surroundings have seemed strange, confusing, threatening or unreal	yes/no	past 30 days
Prodromal Questionnaire - having had the sense that some person or force was around me, even though I could not see anyone	yes/no	past 30 days
Prodromal Questionnaire - having heard unusual sounds like banging, clicking, hissing, clapping or ringing in ears	yes/no	past 30 days
Prodromal Questionnaire - having heard things other people could not hear like voices of people whispering or talking	yes/no	past 30 days
Prodromal Questionnaire - having seen things that other people apparently could not see	yes/no	past 30 days
Prodromal Questionnaire - none of the above	yes/no	

Description item	Description response options	Recall period
OBSESSING SUBSCALE OF THE OBSESSIVE COMPULSIVE INVENTORY REVISED (OCI-R)		
OCI-R - degree of difficulty of controlling own thoughts	5-point Likert-type item: not at all, a little, moderately, a lot, extremely	past 30 days
OCI-R - degree of being upset by unpleasant thoughts that come into mind against will	5-point Likert-type item: not at all, a little, moderately, a lot, extremely	past 30 days
OCI-R - frequency of getting nasty thoughts and having difficulty in getting rid of them	5-point Likert-type item: not at all, a little, moderately, a lot, extremely	past 30 days
ADAPTED PRIME-MD EATING DISORDER ITEMS		
Prime-MD Eating Disorder - number of days in past 30 days with binge eating episodes	0-30	past 30 days
Prime-MD Eating Disorder - number of days in past 30 days with purging behaviors	0-30	past 30 days
TREATMENT USE SECTION		
Psychological help for emotional, substance use problem - ever	yes/no	lifetime
Medication for emotional, substance use problem - ever	yes/no	lifetime
Psychological help for emotional, substance use problem - past 12 months	yes/no	past 12 months
Medication for emotional, substance use problem - past 12 months	yes/no	past 12 months
Psychological help for emotional, substance use problem - since start COVID-9 pandemic	yes/no	since COVID-19 pandemic
Medication for emotional, substance use problem - since start COVID-9 pandemic	yes/no	since COVID-19 pandemic
Psychological help or psychiatric medication interrupted due to COVID-19 pandemic	yes/no	since COVID-19 pandemic
Need for (additional) psychological help or medication for emotional, substance use problem since start COVID-19 pandemic	yes/no	since COVID-19 pandemic
USE OF ONLINE MENTAL HEALTH RESOURCES (AD HOC DEVELOPED)		
Number of days in past 30 days used hospital emergency department services	0-10+	past 30 days
Number of days in past 30 days used primary care emergency services	0-10+	past 30 days
Number of days in past 30 days visited family physician	0-10+	past 30 days
Number of days in past 30 days visited psychiatrist or psychologist	0-10+	past 30 days
Number of days in past 30 days used occupational health services	0-10+	past 30 days
Number of days in past 30 days used specific mental health resources offered by workplace	0-10+	past 30 days

Description item	Description response options	Recall period
Amount of time in past 30 days used online self-help programs	less than 30 minutes, 30 minutes - 1 hour, 2 - 3 hours, 4 - 5 hours, 6 - 10 hours, 11 hours or more	past 30 days
USE OF PSYCHOTROPIC DRUGS		
30-day use of psychotropic medication	yes/no	past 30 days
30-day use of antidepressants	yes/no	past 30 days
30-day use of anxiolytics	yes/no	past 30 days
30-day use of hypnotics	yes/no	past 30 days
30-day use of stimulants	yes/no	past 30 days
30-day use of mood stabilizers	yes/no	past 30 days
30-day use of antipsychotics	yes/no	past 30 days
BARRIERS TO SEEK MENTAL HEALTH TREATMENT		
Stages of Change Readiness and Treatment Eagerness (SOCRATES) Scale	5 response options: I don't have a problem that I need to change, I have a problem, but I'm not sure I want to take any steps to change it, I have a problem and want to do something about it, I have a problem and I am already actively working to change it, I had a problem, but I've done something about it and things are going better now	current
Barrier for seeking treatment - long waiting lists	5-point Likert-type item: unimportant, of little importance, moderately important, important, very important	current
Barrier for seeking treatment - professional treatment not being available	5-point Likert-type item: unimportant, of little importance, moderately important, important, very important	current
Barrier for seeking treatment - not sure if available treatments are very effective	5-point Likert-type item: unimportant, of little importance, moderately important, important, very important	current
Barrier for seeking treatment - too ashamed	5-point Likert-type item: unimportant, of little importance, moderately important, important, very important	current
Barrier for seeking treatment - unsure of where to go or who to see	5-point Likert-type item: unimportant, of little importance, moderately important, important, very important	current
Barrier for seeking treatment - problems with time, transportation, or scheduling	5-point Likert-type item: unimportant, of little importance, moderately important, important, very important	current
Barrier for seeking treatment - other	5-point Likert-type item: unimportant, of little importance, moderately important, important, very important	current
COVID-19 & WORK SECTION		
CURRENT EMPLOYMENT SITUATION		
Pre-pandemic monthly income	12 response options ranging from less than 570 euro to 6000 euro or more	before COVID-19 pandemic

Description item	Description response options	Recall period
Significant loss in your personal or family's income due to the coronavirus pandemic	yes/no	since COVID-19 pandemic
Frequency working outside home since start COVID-19 pandemic	5-point Likert type item: never, rarely, sometimes, often, almost always, always	during lockdown
Average number of people with whom close contact when working outside of home	0-999	during lockdown
Frequency working from home since start COVID-19 pandemic	5-point Likert type item: never, rarely, sometimes, often, almost always, always	during lockdown
Average weekly hours worked	0-168	during lockdown
Essential worker	yes/no	during lockdown
Healthcare profession	medical doctor, nurse, auxiliary nurse, other profession involved in patient care, other profession not involved in patient care	current
Type of workplace	hospital ED, hospital but not ED, primary care, other	current
Working in private or public healthcare sector, or both	private, public, both	current
Relocated to other workplace because of COVID-19	no changes in assigned functions, team or work location, changed of team or assigned functions	since COVID-19 pandemic
Belief job is posing a serious risk	5-point Likert type item: never, rarely, sometimes, often, almost always, always	since COVID-19 pandemic
Feeling more stress at work	5-point Likert type item: never, rarely, sometimes, often, almost always, always	since COVID-19 pandemic
Feeling like doing tasks for which not trained	5-point Likert type item: never, rarely, sometimes, often, almost always, always	since COVID-19 pandemic
Considering quitting job because of the coronavirus	5-point Likert type item: never, rarely, sometimes, often, almost always, always	since COVID-19 pandemic
People avoid family because of job	5-point Likert type item: never, rarely, sometimes, often, almost always, always	since COVID-19 pandemic
Perceived lack of coordination	5-point Likert type item: never, rarely, sometimes, often, almost always, always	since COVID-19 pandemic
Perceived lack of effective coordination	5-point Likert type item: never, rarely, sometimes, often, almost always, always	since COVID-19 pandemic
Perceived staff shortage	5-point Likert type item: never, rarely, sometimes, often, almost always, always	since COVID-19 pandemic
Perceived lack of supervision	5-point Likert type item: never, rarely, sometimes, often, almost always, always	since COVID-19 pandemic
Perceived frequency of lack of protective equipment	response options always, almost always, sometimes, only once, never, not applicable	since COVID-19 pandemic
Considers available protective equipment sufficient to prevent contagion	response options completely insufficient, very insufficient, somewhat insufficient, sufficient, not applicable	since COVID-19 pandemic
Frequency of direct exposure to patients with COVID-19 infection	5-point Likert type item: never, rarely, sometimes, often, almost always, always	since COVID-19 pandemic

Description item	Description response options	Recall period
Having to prioritize care among COVID-19 patients	yes/no	since COVID-19 pandemic
Degree of distress related to having to prioritize care among COVID-19 patients	5-point Likert type item: no distress, some distress, a lot of distress, a great deal of distress	since COVID-19 pandemic
Having had close contact with someone who has died from COVID-19 because of work	yes/no	since COVID-19 pandemic
Having patient(s) in care that died from COVID-19 infection	yes/no	since COVID-19 pandemic
COVID-19 & ISOLATION, QUARANTINE AND CONFINEMENT SECTION		
Living situation	home, apartment, nursing home, student residence, other	current
Number of elderly or disabled individuals in care at home	0-9	current
Having a terrace or garden	yes/no	current
PSYCHOLOGICAL FUNCTIONING SECTION		
ADAPTED PERI LIFE EVENTS SCALE		
Stress related to concern about possibly being infected with coronavirus	5-point Likert type item: very intense, intense, moderate, mild, none	current
Stress related to concern about loved ones being infected with coronavirus	5-point Likert type item: very intense, intense, moderate, mild, none	current
Stress related to death of a loved one due to coronavirus	5-point Likert type item: very intense, intense, moderate, mild, none	current
Stress related to loss of job or income due to coronavirus	5-point Likert type item: very intense, intense, moderate, mild, none	current
stress related to negative or alarming messages about coronavirus in the media	5-point Likert type item: very intense, intense, moderate, mild, none	current
Stress related to personal health	5-point Likert type item: very intense, intense, moderate, mild, none	current
Stress related to health of loved ones	5-point Likert type item: very intense, intense, moderate, mild, none	current
Stress related to romantic life	5-point Likert type item: very intense, intense, moderate, mild, none	current
Stress related to relationship with family	5-point Likert type item: very intense, intense, moderate, mild, none	current
Stress related to other problems that loved ones have	5-point Likert type item: very intense, intense, moderate, mild, none	current
Stress related to financial situation	5-point Likert type item: very intense, intense, moderate, mild, none	current
Stress related to problems getting along with coworkers	5-point Likert type item: very intense, intense, moderate, mild, none	current
Stress related to life in general	5-point Likert type item: very intense, intense, moderate, mild, none	current

Description item	Description response options	Recall period
TWELVE-MONTH STRESSFUL EXPERIENCES		
12-month stressful event - close friend or family member suffered serious illness, injury, or assault	yes/no	past 12-months
12-month stressful event - close friend of the family or another relative has died	yes/no	past 12-months
12-month stressful event - involved in accident in which own life or lives of others were in danger	yes/no	past 12-months
12-month stressful event - physically assaulted	yes/no	past 12-months
12-month stressful event - sexually assaulted or raped	yes/no	past 12-months
12-month stressful event - none of these problems	yes/no	past 12-months
CONNOR-DAVIDSON RESILIENCE SCALE (CD-RISC)		
CD-RISC - able to adapt when changes occur	5-point Likert type item: never, rarely, sometimes, often, almost always	current
CD-RISC - can deal with whatever comes my way	5-point Likert type item: never, rarely, sometimes, often, almost always	current
CD-RISC - try to see humorous side of things when faced with problems	5-point Likert type item: never, rarely, sometimes, often, almost always	current
CD-RISC - having to cope with stress can make me stronger	5-point Likert type item: never, rarely, sometimes, often, almost always	current
CD-RISC - tend to bounce back after illness, injury or other hardships	5-point Likert type item: never, rarely, sometimes, often, almost always	current
CD-RISC - believe can goals, even if there are obstacles	5-point Likert type item: never, rarely, sometimes, often, almost always	current
CD-RISC - stay focused and think clearly under pressure	5-point Likert type item: never, rarely, sometimes, often, almost always	current
CD-RISC - not easily discouraged by failure	5-point Likert type item: never, rarely, sometimes, often, almost always	current
CD-RISC - think of myself as strong person when dealing with life's challenges and difficulties	5-point Likert type item: never, rarely, sometimes, often, almost always	current
CD-RISC - able to handle unpleasant or painful feelings like sadness, fear, and anger	5-point Likert type item: never, rarely, sometimes, often, almost always	current
OPTIMAL STRUCTURES FOR A MENTALLY HEALTHY LIFE SCALE (AD HOC DEVELOPED)		
Healthy habits - maintain healthy sleep habits	5-point Likert type item: never, less than 1 day a week, 1-2 days a week, 3-4 days a week, every day or almost every day	during lockdown
Healthy habits - maintain a healthy diet	5-point Likert type item: never, less than 1 day a week, 1-2 days a week, 3-4 days a week, every day or almost every day	during lockdown
Healthy habits - exercise for 30 minutes or more	5-point Likert type item: never, less than 1 day a week, 1-2 days a week, 3-4 days a week, every day or almost every day	during lockdown

Description item	Description response options	Recall period
Healthy habits - engage in hobbies or relaxing activities	5-point Likert type item: never, less than 1 day a week, 1-2 days a week, 3-4 days a week, every day or almost every day	during lockdown
Healthy habits - maintain a regular daily routine	5-point Likert type item: never, less than 1 day a week, 1-2 days a week, 3-4 days a week, every day or almost every day	during lockdown
Healthy habits - balance time between work, personal life, and leisure	5-point Likert type item: never, less than 1 day a week, 1-2 days a week, 3-4 days a week, every day or almost every day	during lockdown
QUALITY OF LIFE SECTION		
OSLO SOCIAL SUPPORT SCALE (OSSS-3)		
OSS3 - number of close people to count on if serious problems	none, one or two people, three to five people, six people or more	current
OSS3 - degree of interest people in surroundings show in what happens to you or what you do	5-point Likert type item: none, little, uncertain, some, a lot	current
OSS3 - degree of easiness to get help from neighbors if needed	5-point Likert type item: very difficult, difficult, possible, easy, very easy	current
Degree of letting someone in your surroundings know when having a problem or concern	5-point Likert type item: never, seldom, sometimes, most of the time, always	current
UCLA THREE-ITEM LONELINESS SCALE		
UCLA 1 - frequency of lacking companionship	3-point Likert type item: hardly ever, some of the time, often	during lockdown
UCLA 2 - frequency of feeling left out	3-point Likert type item: hardly ever, some of the time, often	during lockdown
UCLA 3 - frequency of feeling isolated from others	3-point Likert type item: hardly ever, some of the time, often	during lockdown
SOCIAL MEDIA USE ITEMS (AD HOC DEVELOPED)		
Frequency speaking directly with family or friends through video or audio calls	5-point Likert type item: every day or almost every day, 3-4 days a week, 1-2 days a week, less than 1 day a week, never	during lockdown
Frequency exchanging text messages with family or friends	5-point Likert type item: every day or almost every day, 3-4 days a week, 1-2 days a week, less than 1 day a week, never	during lockdown
Degree of importance of social networks to stay in touch with family or friends	5-point Likert type item: every day or almost every day, 3-4 days a week, 1-2 days a week, less than 1 day a week, never	during lockdown
BRIEF ASSESSMENT OF FAMILY FUNCTIONING SCALE (BAFFS)		
BAFFS – ability to express feelings towards each other	4-point Likert-type item: totally agree, agree, disagree, totally disagree	during lockdown
BAFFS – not getting along well together	4-point Likert-type item: totally agree, agree, disagree, totally disagree	during lockdown
BAFFS – confiding in each other	4-point Likert-type item: totally agree, agree, disagree, totally disagree	during lockdown

Description item	Description response options	Recall period
SELECTED ITEMS FROM THE PARENTAL STRESS SCALE (PSS)		
PSS - major source of stress in life is child(ren)	5-point Likert type item: totally agree, agree, undecided, disagree, totally disagree	during lockdown
PSS - having child(ren) leaves little time and flexibility in life	5-point Likert type item: totally agree, agree, undecided, disagree, totally disagree	during lockdown
PSS - having child(ren) has been a financial burden	5-point Likert type item: totally agree, agree, undecided, disagree, totally disagree	during lockdown
PSS - difficult to balance different responsibilities because of child(ren)	5-point Likert type item: totally agree, agree, undecided, disagree, totally disagree	during lockdown
FIVE-LEVEL VERSION OF EQ-5D (EQ-5D-5L)		
EQ-5D-5L item 1 - problems with mobility	5 response options: no/slight/moderate/severe problems in walking about, unable to walk about	today
EQ-5D-5L item 2 - problems with self-care	5 response options: no/slight/moderate/severe problems in washing/dressing oneself, unable to wash/dress oneself	today
EQ-5D-5L item 3 - problems with usual activities	5 response options: no/slight/moderate/severe problems in doing usual activities, unable to do usual activities	today
EQ-5D-5L item 4 - problems with pain or discomfort	5 response options: no/slight/moderate/severe/extreme pain or discomfort	today
EQ-5D-5L item 5 - problems with anxiety or depression	5 response options: no/slightly/moderately/severely/extremely anxiety or depression	today
EQ-5D-5L item 5 - overall health rating	5-point Likert type item: excellent, very good, good, fair, poor	today
SELF-ADMINISTERED COMORBIDITY QUESTIONNAIRE (SCQ)		
SCQ - respiratory disease (not provoked by COVID-19)	yes/no	current
SCQ - cardiovascular disease	yes/no	current
SCQ - diabetes	yes/no	current
SCQ - cancer	yes/no	current
SCQ - chronic liver disease	yes/no	current
SCQ - immunological problems	yes/no	current
SCQ - other medical problems	yes/no	current
SCQ - no problems	yes/no	current
SHEEHAN DISABILITY SCALES		
Sheehan Disability Scale - home management/chores	Visual Analogue Scale 0-10	12 months
Sheehan Disability Scale - work	Visual Analogue Scale 0-10	12 months
Sheehan Disability Scale - close personal relationships	Visual Analogue Scale 0-10	12 months

Description item	Description response options	Recall period
Sheehan Disability Scale - social life	Visual Analogue Scale 0-10	12 months

Supplementary Figure 1. Area under the receiver operating characteristic curve (AUROC) (blue) and area under the precision-recall curve (orange) of each of the Linear Support Vector Classifier (SVC) hyperparameters values.

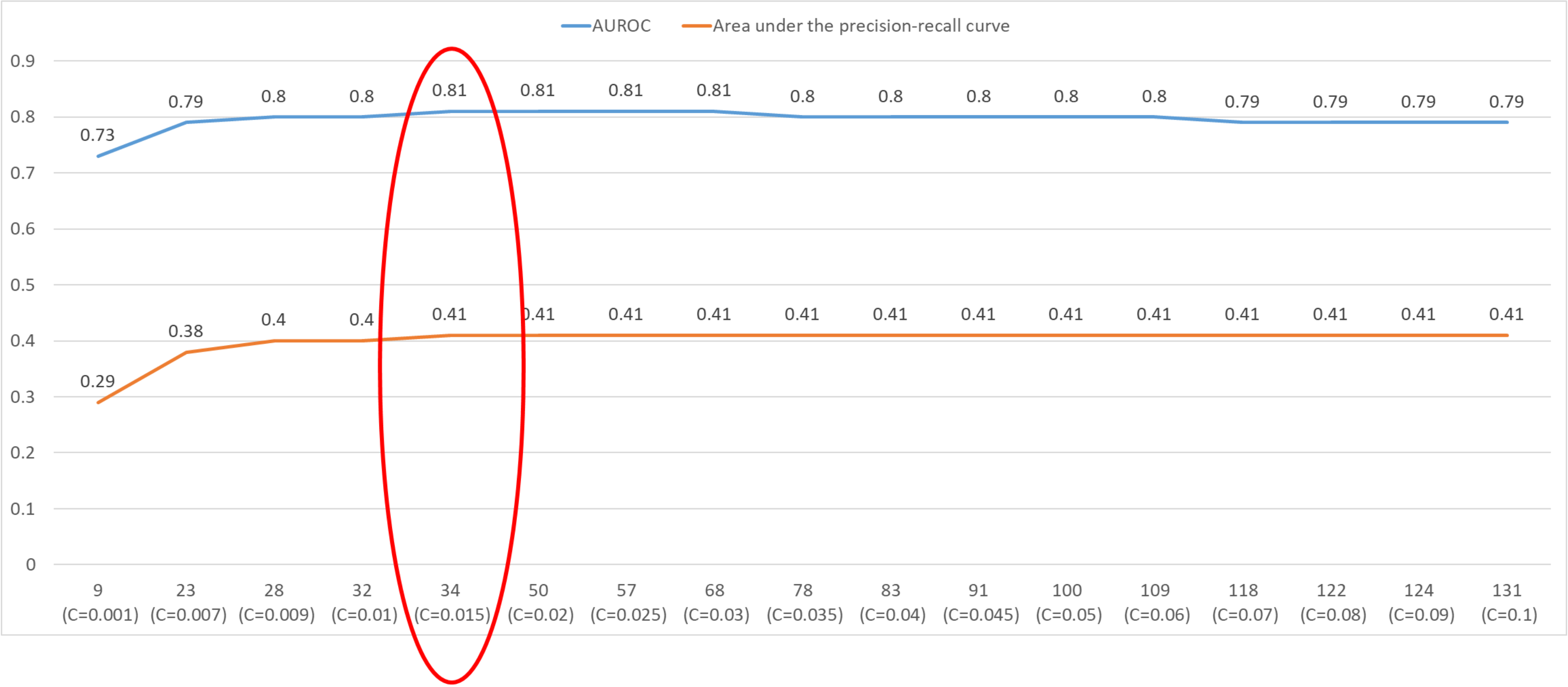

This Figure shows the number of selected variables for each value of hyperparameters C (x-axis). These values represent the average of the number of selected variables in each of the 12 imputations. Additionally, each data point on the graph represents: in blue the area under the ROC curve and in orange area under the precision-recall curve.

Supplementary Table2. The percentage of missing for each variable.

Description item	Percentage of missing data
SOCIODEMOGRAPHIC VARIABLES SECTION	
Age	0.0
Gender	0.0
Country of birth	1.0
Nationality	2.1
Living with partner	0.2
Marital status	0.1
Highest level of education	0.2
Having children in care	0.1
COVID-19 INFECTION STATUS SECTION	
Positive test or medical diagnosis COVID-19	0.3
Number of days hospitalized for COVID-19	0.3
Having been hospitalized for COVID-19	0.3
Ever admitted to intensive care unit for COVID-19	0.3
Belief to have been infected with COVID-19	0.5
Having close ones infected with COVID-19	0.2
Severity of infection of close one most severely infected	0.2
Number of days isolated, in quarantine since start of COVID-19 pandemic	0.2
Having been isolated or in quarantine because of COVID-19	0.1
ADAPTED COVID-19 PERCEIVED RISK SCALE	
Feeling like having little control over getting infected or not	0.0
Thinking that it would be unlikely to survive if having coronavirus.	0.1
Being afraid of infecting loved ones with coronavirus.	0.0
Family and friends are worried about getting infected through me	0.2
COVID-19 GOVERNMENT MEASURES	
Feeling there has been too much concern about COVID-19 pandemic	0.2
MENTAL HEALTH SECTION	
CIDI 3.0 LIFETIME DISORDER SCREENING QUESTIONS	
CIDI 3.0 - reporting prepandemic depression	1.1
CIDI 3.0 - reporting prepandemic bipolar disorder	1.1
CIDI 3.0 - reporting prepandemic panic attacks,disorder	1.1
CIDI 3.0 - reporting prepandemic anxiety issues	1.1
CIDI 3.0 - reporting prepandemic alcohol use problems,disorders	1.1
CIDI 3.0 - reporting prepandemic drug use problems,disorders	1.1
CIDI 3.0 - reporting any other serious prepandemic psychological problem	1.1
CIDI 3.0 - no prepandemic psychological problems	1.1
PATIENT HEALTH QUESTIONNAIRE (PHQ-8)	
PHQ-8 item 1: little interest or pleasure in doing things	0.1
PHQ-8 item 2: feeling down, depressed, or hopeless	0.1
PHQ-8 item 3: trouble falling or staying asleep, or sleeping too much	0.1

PHQ-8 item 4: feeling tired or having little energy	0.1
PHQ-8 item 5: poor appetite or overeating	0.1
PHQ-8 item 6: feelings of low self-esteem or failure	0.2
PHQ-8 item 7: trouble concentrating on things	0.1
PHQ-8 item 8: psychomotor agitation or retardation	0.1
GENERAL ANXIETY DISORDER 7 ITEM SCALE (GAD-7)	
GAD-7 item 1: feeling nervous, anxious, or on edge	0.0
GAD-7 item 2: not being able to stop or control worrying	0.1
GAD-7 item 3: worrying too much about different things	0.1
GAD-7 item 4: trouble relaxing	0.1
GAD-7 item 5: being restless or feeling keyed up or on edge	0.1
GAD-7 item 6: becoming easily annoyed or irritable	0.1
GAD-7 item 7: feeling afraid as if something awful might happen	0.1
PTSD CHECKLIST FOR DSM-5 (PCL-5) – 4 ITEMS	
PCL-5 - intrusion: suddenly feeling or acting as if the stressful experience were actually happening again	0.1
PCL-5 - avoidance: avoiding external reminders of the stressful experience	0.1
PCL-5 - negative alterations in cognition and mood: feeling distant or cut off from other people	0.1
PCL-5 - alterations in arousal and reactivity: irritable behavior, angry outbursts, or acting aggressively	0.1
PANIC ATTACKS - CIDI SCREENING SCALE	
CIDI Screening Scale - number of panic attacks	1.9
CAGE-AID QUESTIONNAIRE	
CAGE-AID - cutting down: felt like should cut down drinking or drug use	1.6
CAGE-AID - annoyance by others: being annoyed by people criticizing drinking or drug use	1.9
CAGE-AID - feelings of guilt: feeling guilty about drinking or drug use	2.0
CAGE-AID - dependency: needed drink, drugs first thing in the morning (eye-opener)	1.5
SUICIDAL THOUGHTS AND BEHAVIORS SCREEN (CSSRS – SITBI)	
C-SSRS - passive suicidal ideation	0.3
C-SSRS - active suicidal ideation	0.3
C-SSRS - number of past 30 days with suicidal ideation (passive, active)	0.4
C-SSRS - suicide plan	0.3
C-SSRS - number of past 30 days with suicide plan	0.4
C-SSRS - suicide attempt	0.3
PERSONAL BURNOUT SUBSCALE OF THE COPENHAGEN BURNOUT INVENTORY (CBI)	
CBI burnout subscale - frequency feeling tired	16.3
CBI burnout subscale - frequency feeling physically exhausted	16.3
CBI burnout subscale - frequency feeling emotionally exhausted	16.3
CBI burnout subscale - frequency thinking: i can’t take it anymore	16.3
CBI burnout subscale - frequency feeling worn out	16.3
CBI burnout subscale - frequency feeling feel weak and susceptible to illness	16.3
SELECTED ITEMS FROM THE PRODROMAL QUESTIONNAIRE	
Prodromal Questionnaire - previously familiar surroundings have seemed strange, confusing, threatening or unreal	17.3
Prodromal Questionnaire - having had the sense that some person or force was around me, even though I could not see anyone	17.3
Prodromal Questionnaire - having heard unusual sounds like banging, clicking, hissing, clapping or ringing in ears	17.3

Prodromal Questionnaire - having heard things other people could not hear like voices of people whispering or talking	17.3
Prodromal Questionnaire - having seen things that other people apparently could not see	17.3
Prodromal Questionnaire - none of the above	17.3
OBSESSING SUBSCALE OF THE OBSESSIVE COMPULSIVE INVENTORY REVISED (OCI-R)	
OCI-R - degree of difficulty of controlling own thoughts	17.0
OCI-R - degree of being upset by unpleasant thoughts that come into mind against will	16.9
OCI-R - frequency of getting nasty thoughts and having difficulty in getting rid of them	17.2
ADAPTED PRIME-MD EATING DISORDER ITEMS	
Prime-MD Eating Disorder - number of days in past 30 days with binge eating episodes	17.1
Prime-MD Eating Disorder - number of days in past 30 days with purging behaviors	16.9
TREATMENT USE SECTION	
Psychological help for emotional, substance use problem - ever	1.1
Medication for emotional, substance use problem - ever	1.1
Psychological help for emotional, substance use problem - past 12 months	2.0
Medication for emotional, substance use problem - past 12 months	1.7
Psychological help for emotional, substance use problem - since start COVID-9 pandemic	2.1
Medication for emotional, substance use problem - since start COVID-9 pandemic	1.9
Psychological help or psychiatric medication interrupted due to COVID-19 pandemic	0.3
Need for (additional) psychological help or medication for emotional, substance use problem since start COVID-19 pandemic	2.5
USE OF ONLINE MENTAL HEALTH RESOURCES (AD HOC DEVELOPED)	
Number of days in past 30 days used hospital emergency department services	15.5
Number of days in past 30 days used primary care emergency services	15.5
Number of days in past 30 days visited family physician	15.4
Number of days in past 30 days visited psychiatrist or psychologist	15.5
Number of days in past 30 days used occupational health services	15.5
Number of days in past 30 days used specific mental health resources offered by workplace	15.4
Amount of time in past 30 days used online self-help programs	16.9
USE OF PSYCHOTROPIC DRUGS	
30-day use of psychotropic medication	17.7
30-day use of antidepressants	19.0
30-day use of anxiolytics	19.0
30-day use of hypnotics	19.0
30-day use of stimulants	19.0
30-day use of mood stabilizers	19.0
30-day use of antipsychotics	19.0
BARRIERS TO SEEK MENTAL HEALTH TREATMENT	
Stages of Change Readiness and Treatment Eagerness (SOCRATES) Scale	18.2
Barrier for seeking treatment - long waiting lists	18.5
Barrier for seeking treatment - professional treatment not being available	18.5
Barrier for seeking treatment - not sure if available treatments are very effective	18.5
Barrier for seeking treatment - too ashamed	18.5
Barrier for seeking treatment - unsure of where to go or who to see	18.4
Barrier for seeking treatment - problems with time, transportation, or scheduling	18.5

Barrier for seeking treatment - other	19.3
COVID-19 & WORK SECTION	
CURRENT EMPLOYMENT SITUATION	
Pre-pandemic monthly income	7.1
Significant loss in your personal or family's income due to the coronavirus pandemic	0.3
Frequency working outside home since start COVID-19 pandemic	0.1
Average number of people with whom close contact when working outside of home	0.0
Frequency working from home since start COVID-19 pandemic	0.3
Average weekly hours worked	0.4
Essential worker	0.4
Healthcare profession	0.3
Type of workplace	0.7
Working in private or public healthcare sector, or both	0.5
Relocated to other workplace because of COVID-19	0.6
Belief job is posing a serious risk	0.0
Feeling more stress at work	0.1
Feeling like doing tasks for which not trained	0.1
Considering quitting job because of the coronavirus	0.1
People avoid family because of job	0.1
Perceived lack of coordination	0.1
Perceived lack of effective coordination	0.1
Perceived staff shortage	0.1
Perceived lack of supervision	0.1
Perceived frequency of lack of protective equipment	0.3
Considers available protective equipment sufficient to prevent contagion	0.5
Frequency of direct exposure to patients with COVID-19 infection	0.6
Having to prioritize care among COVID-19 patients	0.7
Degree of distress related to having to prioritize care among COVID-19 patients	0.7
Having had close contact with someone who has died from COVID-19 because of work	0.5
Having patient(s) in care that died from COVID-19 infection	0.9
COVID-19 & ISOLATION, QUARANTINE AND CONFINEMENT SECTION	
Living situation	0.0
Number of elderly or disabled individuals in care at home	0.8
Having a terrace or garden	0.1
PSYCHOLOGICAL FUNCTIONING SECTION	
ADAPTED PERI LIFE EVENTS SCALE	
Stress related to concern about possibly being infected with coronavirus	0.0
Stress related to concern about loved ones being infected with coronavirus	0.0
Stress related to death of a loved one due to coronavirus	0.3
Stress related to loss of job or income due to coronavirus	0.2
stress related to negative or alarming messages about coronavirus in the media	0.1
Stress related to personal health	0.1
Stress related to health of loved ones	0.0

Stress related to romantic life	0.1
Stress related to relationship with family	0.1
Stress related to other problems that loved ones have	0.1
Stress related to financial situation	0.1
Stress related to problems getting along with coworkers	0.1
Stress related to life in general	0.1
TWELVE-MONTH STRESSFUL EXPERIENCES	
12-month stressful event - close friend or family member suffered serious illness, injury, or assault	1.1
12-month stressful event - close friend of the family or another relative has died	1.1
12-month stressful event - involved in accident in which own life or lives of others were in danger	1.1
12-month stressful event - physically assaulted	1.1
12-month stressful event - sexually assaulted or raped	1.1
12-month stressful event - none of these problems	1.1
CONNOR-DAVIDSON RESILIENCE SCALE (CD-RISC)	
CD-RISC - able to adapt when changes occur	0.0
CD-RISC - can deal with whatever comes my way	0.0
CD-RISC - try to see humorous side of things when faced with problems	0.0
CD-RISC - having to cope with stress can make me stronger	0.0
CD-RISC - tend to bounce back after illness, injury or other hardships	0.0
CD-RISC - believe can goals, even if there are obstacles	0.0
CD-RISC - stay focused and think clearly under pressure	0.0
CD-RISC - not easily discouraged by failure	0.0
CD-RISC - think of myself as strong person when dealing with life’s challenges and difficulties	0.1
CD-RISC - able to handle unpleasant or painful feelings like sadness, fear, and anger	0.0
OPTIMAL STRUCTURES FOR A MENTALLY HEALTHY LIFE SCALE (AD HOC DEVELOPED)	
Healthy habits - maintain healthy sleep habits	16.7
Healthy habits - maintain a healthy diet	16.7
Healthy habits - exercise for 30 minutes or more	16.7
Healthy habits - engage in hobbies or relaxing activities	16.7
Healthy habits - maintain a regular daily routine	16.7
Healthy habits - balance time between work, personal life, and leisure	16.7
QUALITY OF LIFE SECTION	
OSLO SOCIAL SUPPORT SCALE (OSSS-3)	
OSS3 - number of close people to count on if serious problems	0.0
OSS3 - degree of interest people in surroundings show in what happens to you or what you do	0.0
OSS3 - degree of easiness to get help from neighbors if needed	0.1
Degree of letting someone in your surroundings know when having a problem or concern	0.0
UCLA THREE-ITEM LONELINESS SCALE	
UCLA 1 - frequency of lacking companionship	16.5
UCLA 2 - frequency of feeling left out	16.4
UCLA 3 - frequency of feeling isolated from others	15.9
SOCIAL MEDIA USE ITEMS (AD HOC DEVELOPED)	
Frequency speaking directly with family or friends through video or audio calls	15.9

Frequency exchanging text messages with family or friends	15.9
Degree of importance of social networks to stay in touch with family or friends	16.3
BRIEF ASSESSMENT OF FAMILY FUNCTIONING SCALE (BAFFS)	
BAFFS – ability to express feelings towards each other	17.1
BAFFS – not getting along well together	19.2
BAFFS – confiding in each other	18.5
SELECTED ITEMS FROM THE PARENTAL STRESS SCALE (PSS)	
PSS - major source of stress in life is child(ren)	6.4
PSS - having child(ren) leaves little time and flexibility in life	6.4
PSS - having child(ren) has been a financial burden	6.4
PSS - difficult to balance different responsibilities because of child(ren)	6.4
FIVE-LEVEL VERSION OF EQ-5D (EQ-5D-5L) *	
EQ-5D-5L item 1 - problems with mobility	40.3
EQ-5D-5L item 2 - problems with self-care	40.4
EQ-5D-5L item 3 - problems with usual activities	40.3
EQ-5D-5L item 4 - problems with pain or discomfort	40.3
EQ-5D-5L item 5 - problems with anxiety or depression	40.3
EQ-5D-5L item 5 - overall health rating	2.7
SELF-ADMINISTERED COMORBIDITY QUESTIONNAIRE (SCQ)	
SCQ - respiratory disease (not provoked by COVID-19)	4.0
SCQ - cardiovascular disease	4.0
SCQ - diabetes	4.0
SCQ - cancer	4.0
SCQ - chronic liver disease	4.0
SCQ - immunological problems	4.0
SCQ - other medical problems	4.0
SCQ - no problems	4.0
SHEEHAN DISABILITY SCALES*	
Sheehan Disability Scale - home management/chores	39.7
Sheehan Disability Scale - work	39.7
Sheehan Disability Scale - close personal relationships	39.7
Sheehan Disability Scale - social life	39.7

*60% of the sample was randomly selected to respond to this section.
